# An Asparagine-Rich Protein Nbnrp1 Modulate *Verticillium dahliae* Protein PevD1-Induced Cell Death and Disease Resistance in *Nicotiana benthamian*a

**DOI:** 10.3389/fpls.2018.00303

**Published:** 2018-03-07

**Authors:** Yingbo Liang, Shichun Cui, Xiaoli Tang, Yi Zhang, Dewen Qiu, Hongmei Zeng, Lihua Guo, Jingjing Yuan, Xiufen Yang

**Affiliations:** The State Key Laboratory for Biology of Plant Diseases and Insect Pests, Institute of Plant Protection, Chinese Academy of Agricultural Sciences, Beijing, China

**Keywords:** *Verticillium dahliae*, PevD1, Nbnrp1, protein–protein interaction, defense response, disease resistance

## Abstract

PevD1 is a fungal protein secreted by *Verticillium dahliae*. Our previous researches showed that this protein could induce hypersensitive responses-like necrosis and systemic acquired resistance (SAR) in cotton and tobacco. To understand immune activation mechanisms whereby PevD1 elicits defense response, the yeast two-hybrid (Y2H) assay was performed to explore interacting protein of PevD1 in *Arabidopsis thaliana*, and a partner AtNRP (At5g42050) was identified. Here, AtNRP homolog in *Nicotiana benthamiana* was identified and designated as Nbnrp1. The Nbnrp1 could interact with PevD1 via Y2H and bimolecular fluorescence complementation (BiFC) analyses. Moreover, truncated protein binding assays demonstrated that the C-terminal 132 amino acid (development and cell death, DCD domain) of Nbnrp1 is required for PevD1-Nbnrp1 interaction. To further investigate the roles of Nbnrp1 in PevD1-induced defense response, Nbnrp1-overexpressing and Nbnrp1-silence transgenic plants were generated. The overexpression of Nbnrp1 conferred enhancement of PevD1-induced necrosis activity and disease resistance against tobacco mosaic virus (TMV), bacterial pathogen *Pseudomonas syringae* pv. *tabaci* and fungal pathogen *V. dahliae*. By contrast, Nbnrp1-silence lines displayed attenuated defense response compared with the wild-type. It is the first report that an asparagine-rich protein Nbnrp1 positively regulated *V. dahliae* secretory protein PevD1-induced cell death response and disease resistance in *N. benthamiana*.

## Introduction

Plants have evolved a sophisticated innate immune system to detect and ward off potential dangers in the course of plant-pathogens co-evolution (Dangl and Jones, 2001; Akira et al., 2006; Chisholm et al., 2006; Boller and Felix, 2009). Plant immune system follows two major strategies. The recognition of conserved pathogen or microbe-associated molecular patterns (P/MAMPs) known as microbe signature is the primary layer of the plant immune system, called as PAMP-triggered immunity (PTI). For infecting host plant successfully, pathogens suppress PTI by employing effectors that target or interfere with host defense signaling components. Afterward, plants generated specific recognition system to perceive such effectors, leading to effector-triggered immunity (ETI) (Dodds and Rathjen, 2010). ETI is generally characterized stronger and more sustained immune responses than PTI, whereas PTI represent more durable and broad-spectrum resistance (Katagiri and Tsuda, 2010; Tsuda and Katagiri, 2010). In facts, it is well documented that the distinction between PTI and ETI, between PAMPs and effectors, even between R protein and defense protein is imprecise (Thomma et al., 2011). PAMPs and effectors trigger similar defense responses and converge in common downstream immune signal cascade including hypersensitive response (HR)-like cell death, oxidative burst, activation of kinase signaling cascades, expression of defense-related gene and phytoalexin accumulation, etc. which lead to systemic acquired resistance (SAR) that confers broad spectrum pathogen resistance to bacteria, fungi, and virus disease in plants.

Hypersensitive response is commonly considered as an example of programmed cell death (PCD) and a typical rapid defense response induced by microbe effectors, which alerts neighboring cells and causes rapid plant cell death that lead to restriction of pathogen further spread (Pennell and Lamb, 1997; Heath, 2000; Desaki et al., 2006; Mur et al., 2008). Plant cell death response can be triggered by different mechanisms, in which HR induced by microbe effectors is typically incidental to resistance (Mur et al., 2008; Tsuda and Katagiri, 2010; Zhang et al., 2017a). Given these studies, it’s essential to enrich our understanding of how can microbe effectors activate downstream signal component in HR.

We generally accept that plant directly or indirectly sense elicitor/effectors through pattern recognition receptors (PRRs) localized in the plasma membrane or cytoplasm. Pathogenic fungi secrete hundreds of effectors/PAMPs during the infection process, which modulate the plant-fungus interaction through targeting plant molecular. Identification of elicitor target in plant is vital for understanding the elicitor-activating plant defense signaling cascade. Although many elicitors/effectors have been characterized, the described target partner in plants is limited.

PevD1, a proteinaceous elicitor secreted by *Verticillium dahliae*, could induce typical HR-like necrosis and apoptosis-related events in tobacco (Wang et al., 2011) as well as improve SAR against tobacco mosaic virus (TMV) and cotton *Verticillium* Wilt (Bu et al., 2014). The infiltration of PevD1 elevated the expression level of SAR-related genes of *PR1-a*, *PR1-b*, *NPR1* and defense-related genes of *PAL*, *C4H1*, *4CL*, which involve in phenylpropanoid metabolism pathway, and also systematically elicit H_2_O_2_ production, NO generation, lignin deposition, and vessel reinforcement in cotton plants (Bu et al., 2014). *PevD1*-transgenic *Arabidopsis thaliana* lines have been demonstrated to improve disease resistance against *Botrytis cinerea* and *P.s*. pv. tomato DC3000 compared to wild type (Liu et al., 2016). These data indicate that PevD1 could induce broad spectrum pathogen resistance in host plants. Nevertheless, the underlying molecular mechanism involving in PevD1-induced HR and disease resistance in plants needs further research.

To investigate how PevD1 modulates immunity response in plants, we have identified an asparagine-rich protein (AtNRP) in *A. thaliana* as the interacting partner of PevD1 via Y2H system (Zhou et al., 2017). NRPs (Asparagine-rich proteins or N-rich proteins) were originally characterized for their sequence with high amount of the amino acid asparagine in N-terminal. These proteins also contain a DCD domain in C-terminal, which is conserved and involved in development and cell death in plants. The AtNRP could interact with cryptochrome 2 (CRY2), leading to accumulation of CRY2 in the cytoplasmic. Further investigation suggested PevD1 indirectly activated cryptochrome 2 by antagonizing NRP functions, resulting in early flowering upon *V. dahliae* infection (Ludwig and Tenhaken, 2001; Zhou et al., 2017).

In this study, a *Nicotiana benthamiana* NRP protein designated as Nbnrp1 was homology cloned based on conserved PevD1 partner AtNRP. The interaction of Nbnrp1 and PevD1 was confirmed via Y2H and bimolecular fluorescence complementation (BIFC) assays. Further exploration revealed the C-terminal DCD domain of Nbnrp1 was required for the Nbnrp1-PevD1 interaction. Many evidences showed that asparagine-rich protein was involved in response to different stresses such as oxidative stress, salt stress, pathogen infection and transduce cell death signal through either the endoplasmic reticulum (ER) or osmotic stress (Costa et al., 2008; Reis et al., 2011). To investigate the role of Nbnrp1 in PevD1-induced cell death and disease resistance, we generated *Nbnrp1*-overexpressing and *Nbnrp1*-silence transgenic *N. benthamian*a plants. TMV, *Pseudomonas syringae* pv. *tabaci* and *V. dahliae* were used to compare disease resistance between transgenic plants and wild type plants. The results indicated that *Nbnrp1*-overexpressing plants displayed enhanced necrosis activity and disease resistance, whereas *Nbnrp1*-silence plants showed impaired necrosis activity and disease resistance. Accordingly, we proposed that Nbnrp1 is a positive regulator involved in PevD1-elicited cell death and disease resistance in *N. benthamiana*.

## Materials and Methods

### Yeast, Bacterial, and Plant Culture

The prokaryotic expression vector pGEX-6P-2, the plant expression vector pBI121 and pCAMBIA2300, RNAi vector pRNAi1017 were taken from laboratory stocks. TMV-GFP was a gift from Yule Liu (Tsinghua University, Beijing, China). Y2H gold Yeast (*Saccharomyces cerevisiae*) was grown in PDA or SD medium at 28°C. *Escherichia coli* Trans1-T1 and BL21 were purchased from TransGen Biotech (China) and were grown in LB at 37°C. *Agrobacterium tumefaciens* GV3101 was grown in LB medium at 28°C. *P.s.* pv. *tabaci* was a gift from Jun Liu (Institute of Microbiology, Chinese Academy of Sciences, Beijing, China), and grown in KB medium with Rif at 28°C, 200 rpm in an orbital shaker and harvested at log phase of growth (OD_600_ = 1.0). OD600 = 0.002 of *P.s.* pv. tabaci (1 × 10^6^ CFU.mL^-1^) was used for syringing (Zhang et al., 2015).

Tobacco seeds (*N. benthamiana*) were surface sterilized for 3 min in 75% ethanol, rinsed with sterile water for five times, and then germinated in 1/2 MS medium in a growth chamber maintained at 25°C (14 h light/10 h dark). Following germination, seedlings were transferred to plantlets with autoclaved soil consisting of 1:1 (v/v) high-nutrient soil and vermiculite in pots and then cultured in a growth chamber at 25°C with 50% humidity (14 h light/10 h dark). The plants were watered on alternate days.

### Gene Clone and Yeast Two-Hybrid Analysis

AtNRP protein sequence was used for blast search in *N. benthamiana* genome database^1^ and finally obtained raw sequence of a putative protein (SGN-U514876) that contains 57.7% protein sequence similarity to AtNRP. The coding sequence of the protein was cloned with gene-specific primer set (Supplementary Table S1) and we designated this protein as Nbnrp1.

To investigate the PevD1-Nbnrp1 interaction, *PevD1* and *Nbnrp1* gene were cloned into BD vector of the pGBKT7 and AD vector of pGADT7 respectively. Y2H analysis was performed according to the protocol of the manufacturer (Matchmaker Gold Yeast Two-Hybrid System). The fragment of Nbnrp1ΔC (residues 1–199) and Nbnrp1ΔN (residues 200 to 332) were cloned into pGADT7 vector, respectively. The *pevD1*-PGBKT7 and PGADT7 derivates were co-transformed into yeast competent cells. Transformants were screened for growth on SD medium containing X-α-gal but lacking Leu, His, Trp, and Ade (Jin et al., 2007).

### BiFC Assays

The pSCYNE and pSCYCE plasmids were used for BIFC assays. *PevD1* was cloned into pSCYNE to generate *PevD1*-pSCYNE and *Nbnrp1* was cloned into pSCYCE to generate *Nbnrp1*-pSCYCE (Walter et al., 2004). The constructed *PevD1*-pSCYNE and *Nbnrp1*-pSCYCE plasmids were transformed into *A. tumefaciens* GV3101, respectively, and GV3101 were then infiltrated into the leaves of 4-week-old plants at the same time. The epidermal layers of the leaves were assayed for fluorescence 2 days after infiltration (Waadt et al., 2008). The fluorescence of cyan fluorescent protein (CFP) was examined under a laser confocal microscope (Fluo View 1000, Olympus).

### Protein Preparation

PevD1 was expressed and purified according to a previously described protocol (Wang et al., 2011). Protein concentration was measured using the BCA protein assay kit (Pierce, Rockford, IL, United States). Specific primers were designed to amplify full length of *Nbnrp1*, DCD domain (Nbnrp1ΔN) and N-rich domain (Nbnrp1ΔC) fragments (Supplementary Table S1). These three fragments were digested with *BamH* I and *Sal* I, and then were cloned into pGEX-6p-2 vector, respectively. Recombinant vectors were transformed into *E. coli* BL21. Cells transformed with pGEX-6p-2-Nbnrp1, pGEX-6p-2-Nbnrp1ΔN and pGEX-6p-2-Nbnrp1ΔC were cultured in LB medium containing ampicillin (100 mg.L^-1^) at 37°C, 220 rpm for 8 h. Isopropyl b-D-thiogalactoside (IPTG) was then added to a final concentration of 0.2 mM to induce expression at 16°C for 8 h. A culture transformed with the empty pGEX-6P-2 vector was used as a control. The bacteria were pelleted and re-suspended in buffer I (50 mM Tris and 200 mM NaCl, pH 8.0) and the cells were disrupted via sonication. The disrupted cells were then pelleted, and the supernatant was collected. GST affinity purification technology, desalination and ion exchange chromatography were utilized to purify GST-Nbnrp1, GST-Nbnrp1ΔN, GST-Nbnrp1ΔC and GST. All the samples were subjected to sodium dodecyl sulfate-polyacrylamide gel electrophoresis (SDS-PAGE) analysis (Zhang et al., 2017c).

### Pull-Down Assays

6 × His tagged PevD1 and GST, GST-Nbnrp1, GST-Nbnrp1ΔN, GST-Nbnrp1ΔC expressed and purified from *E. coli* were used for GST pull-down assay. 6 × His tagged PevD1 and GST-fused protein were mixed with the GST affinity at 4°C for 4 h. The GST affinity tag was washed with buffer (PBS) 3–5 times and then with the elution buffer (PBS, 20 mM GSH). The eluate was separated via 15% SDS-PAGE and transferred to a polyvinylidene fluoride (PVDF) membrane (Bio-Rad) using a Trans-Blot SD semi-dry electrophoretic transfer cell (Bio-Rad). Western blot was utilized to analyze the results. Proteins were probed with primary antibody anti-His tag mouse monoclonal antibody and anti-GST tag mouse monoclonal antibody (Abbkine) followed by secondary antibody alkaline phosphatase-conjugated goat anti-rabbit IgG (Transgene). The membrane was visualized with a 1 mL BCIP/NBT solution (Transgene) and observed the results using Tanon 5200 chemiluminescence apparatus (Beijing Yuanpinghao Biotech) (Sambrook and Russell, 2004; Shi et al., 2017).

### Generation of Nbnrp1-Overexpressing and Nbnrp1 Silence Transgenic Tobacco Plants

The coding sequence of *Nbnrp1* was amplified by PCR using specific primers PBI121-Nbnrp1-F/R (Supplementary Table S1) and inserted into pBI121 to construct the fusion vector pBI121-*Nbnrp1*. The *Nbnrp1* fragment and the expression vector pBI121 were digested with *BamH* I, and then the *Nbnrp1* fragment was ligated into pBI121 vector using T4 ligase. The final overexpression vector, pBI121-Nbnrp1, containing the Nos terminator, NPT-II gene, and the CaMV35S promoter, was then transformed into *N. benthamiana.*

The sense fragment of *Nbnrp1* was amplified by PCR using primers Sense-F/R (Supplementary Table S1), digested with *Bgl* II and constructed into the pRNAi1017 vector to get pRNAi1017-S vector. The anti-sense fragment of Nbnrp1 was amplified by PCR using primers Anti-sense-F/R (Supplementary Table S1), and digested with *Sal* I and *BamH* I, and then ligated into the pRNAi1017-S vector to construct pRNAi1017-SA vector. Then the pCAMBIA2300 vector and the pRNAi1017-SA vector were digested with *Sal* I and *Pst* I, and the fragment with anti-sense and sense fragment of Nbnrp1 and intron was inserted into pCAMBIA2300 to construct the fusion vector pCAMBIA2300-pRNAi1017-*Nbnrp1*. The plasmid was subsequently transformed into *Agrobacterium* GV3101. *Agrobacterium* GV3101 harboring fusion vector was then transformed into *N. benthamiana* according to the method proposed by Horsch (1985).

1/2 MS medium with Kanamycin (30 mg/L) was used to select for positive transformants and PCR analysis was performed to detect putative transgenic tobacco plants. Genomic DNA from fresh, fully expanded tobacco leaves was extracted and PCR analysis was conducted using a specific fragment of the *NTP II* gene as a primer. After identification of positive transgenic plants, Southern blotting was performed to determine the copy numbers of *Nbnrp1* according to the protocol provided with the DIG High Prime DNA Labeling and Detection Starter Kit II (Amersham Biosciences). To confirm that the selected transgenic lines were overexpression lines or with high silence efficiency, qPCR were performed using RNA prepared from transgenic plants. Non-transgenic plants served as controls. T_3_ homozygous plants were used in this study.

### PevD1-Mediated Cell Death and Pathogen Inoculation Assay

In cell death assay, 4-week-old *N. benthamiana* leaves were infiltrated with 50 μl PevD1 solution in a gradient of concentrations (0.2, 0.5, 1.0, 2, and 5 μM) and the buffer was used as control. The resultant necrotic lesions were observed at 12, 24, and 48 h after treatment (Zhang et al., 2017b). TMV-GFP is a recombinant virus in which the jellyfish GFP gene in inserted into the coat protein (CP) open reading frame (ORF) of native TMV. GFP was visualized using a 100 W long-wave UV lamp (Black Ray model B 100AP; UVP, Upland, CA, United States). Recombination did not influence infection and movement of the virus in *N. benthamiana* (Liu et al., 2002).

*Nicotiana benthamiana* (Nbnrp1-overexpressing, wild type and Nbnrp1-silence lines) were infiltrated with 30 μl of 10 μM PevD1. BSA was used as negative control. Three days later, the upper three untreated leaves were inoculated with the TMV-GFP solution. The number of TMV-GFP lesions on each leaf was counted at 3 days post-inoculation (dpi), as previously described (Wang et al., 2011). The inhibition of TMV-GFP lesions was calculated using the following formula: inhibition (%) = [(number of lesions on wild-type plants - number of lesions on *Nbnrp1*-overexpressing plants)/number of lesions on wild-type plants] × 100.

For bacteria bioassay, the upper three PevD1-untreated leaves were inoculated with 50 μL bacterial suspension of *P. s.* pv. tabaci at 3 days post PevD1 treatment. The plants were maintained at a constant humidity for 3 days. Whole leaves were detached from the host plant and placed in 70% ethanol solution for 1 min, then rinsed in sterile distilled water three times. 1.5 cm^2^ leaf disks were excised from the sampled leaves and ground with a plastic pestle in a 1.5 mL microfuge tube with 100 μL of sterile distilled water. A serial 1:10 (100 μL: 900 μL) dilution series was generated for each sample. The samples were plated on KB medium (Rif) and kept at 28°C for 48 h. The bacterial colonies obtained from each dilution of each sample were counted and analyzed.

Assays for the pathogenicity of *V. dahliae* were performed as described (Bu et al., 2014; Zhang et al., 2017a). *V. dahliae* was cultured on potato dextrose agar medium at 28°C in the dark for 5 days, and then the fungus was transferred into Czapek’s medium with shock culturing at 150 rpm for 2–3 days at 25°C in the dark. The conidial suspension was adjusted to 10^7^ order magnitude using sterile distilled water for inoculation. Four-week-old *N. benthamiana* were treated with 30 μl of 10 μM PevD1 on cotyledons using a 1 ml syringe without needles. BSA was used as negative control. The inoculation of *V. dahliae* was conducted as described below: 20 ml of the conidial suspension was poured into each pot until complete absorption. Every experiment was performed with 24 plants and replicated three times. The inoculated seedlings were then grown for 14 days at 25°C, with a day/night period of 14/10 h. The degree of wilt disease was divided into six grades: 0—healthy plant; grade 1—yellowing cotyledons; grade 2—wilting of one third of the leaves; grade 3—wilting of two thirds of the leaves; grade 4—wilting of all leaves; grade 5—plant death. Disease index value = [Σ(the number of seedling of every grade × relative grade)/total seedlings × highest score (4)] × 100 (Zhang et al., 2017a).

### RT-PCR and Quantitative Real-Time PCR

To investigate the transcription of defense-related genes, *Nbnrp1*-overexpressing lines, *Nbnrp1*-silence lines and wild type plant leaves were infiltrated with 10 μM PevD1. The samples were collected from the upper leaves at the indicated times and then rapidly frozen in liquid nitrogen. Total RNA was extracted with the RNA prep pure Plant Kit (TIANGEN Biotech). Residual genomic DNA was eliminated by treatment with a gDNA Eraser. First-strand cDNA was synthesized from 100 ng of total RNA using reverse transcriptase (TIANGEN Biotech) according to the supplier’s protocol. Quantitative Real-time quantitative PCR (qPCR) was performed to determine the relative expression levels of several defense-related genes using SYBR Green PCR Master Mix (TIANGEN Biotech). Specific genes primers were designed according to the coding sequences of each gene using Beacon Designer 8. PCR mixture was processed on a Bio-Rad CFX Manager (Bio-Rad). Three technical replicates were amplified for each sample, including negative controls. *EF-1a* was used as an internal standard. Quantification of the relative changes in gene transcript levels was performed using the 2^-ΔΔC_T_^ method (Dean et al., 2002; Schmittgen and Livak, 2008).

### Statistical Analysis

All experiments and data presented here involved at least three repeats. The data are presented as means and standard deviations. The statistical analysis was performed with Statistical Analysis System (SAS) software using Student’s *t*-test.

## Results

### Identification of the PevD1 Interacting Protein Nbnrp1

Elicitor PevD1 has been shown to induce broad spectrum resistance in various plants including cotton, tobacco, and *Arabidopsis*. Accordingly, partner protein of PevD1 should be conserved in plants. As NRPs are widely distributed and highly conserved among plants, we cloned AtNRP homologous gene from *N. benthamiana*, designated as Nbnrp1.

**FIGURE 1:**
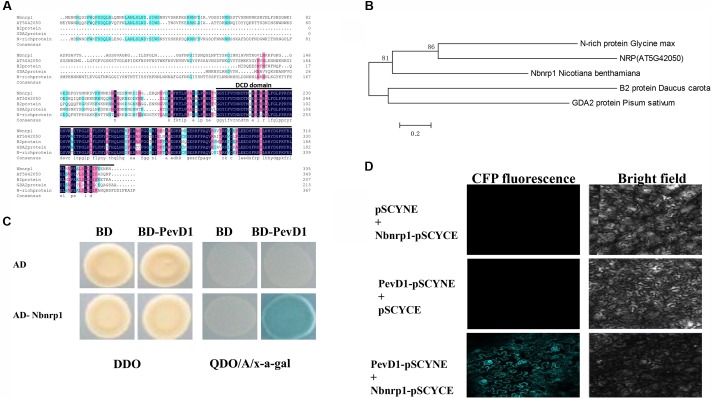
Structural analysis and phylogenetic relationships of Nbnrp1 homologs and interaction of PevD1 and Nbnrp1. **(A)** Alignment of Nbnrp1 homologs in some plants. Consensus represents conserved amino acid residues, black line highlighted areas show DCD domain. **(B)** Phylogenetic analysis of Nbnrp1 and its homologs. **(C)** Interaction of PevD1 and Nbnrp1 in yeast. The indicated AD and BD constructs were transformed into the Y2H gold yeast strain. Transformants were assayed for the activity of protein–protein interactions using reporter genes based on growth on QDO/A/x-α-gal selective medium (SD/-Ade/-His/-Leu/-Trp medium containing Aureobasidin A and x-α-gal). DDO (SD/-Leu/-Trp medium) was used to observe the growth of transformants on non-selective control plates. **(D)** BiFC visualization of the interaction between PevD1 and Nbnrp1 in tobacco leaves. Scale bar = 50 μm. CFP fluorescence and bright field images of leaf cells from *Nicotiana benthamiana* infiltrated with a mixture of *Agrobacterium* suspensions harboring constructs encoding the indicated proteins.

Bioinformatics analysis showed that Nbnrp1 contains 396 amino acid residues, has a predicted molecular mass of 37 kDa, and includes 2 domains (an asparagine-rich domain and a DCD domain). Nbnrp1 was characterized as an asparagine-rich protein because of a high content of the amino acid asparagine (about 20%) in its N-terminus. Nbnrp1 belongs to subgroup of the DCD protein family according to the location of the DCD domain in the protein (Tenhaken et al., 2005). Thus far, the functions of DCD protein have been described in only four homologs, including the B2-protein from carrot (Schrader et al., 1997), GDA2 identified in pea (Li et al., 1998), N-rich protein isolated in soybean (Ludwig and Tenhaken, 2001) and AtNRP that was found from *Arabidopsis* previously (Zhou et al., 2017). The amino acid sequence of Nbnrp1 showed 46.27, 40.65, 55.38, and 57.7% similarity with B2, GDA2, N-rich protein, and AtNRP, respectively. Sequence alignment of Nbnrp1 and its homologs indicated the conserved DCD domain (**Figure 1A**). In an unrooted phylogenetic tree (**Figure 1B**), Nbnrp1 shares high similarity with N-rich protein and AtNRP. To investigate whether Nbnrp1 could bind with PevD1, the Y2H assay was carried out. The BD vector with the PevD1 without the signal peptide sequence and AD vector with Nbnrp1 were constructed and co-transformed into yeast cell. Co-transformed BD/AD, BD/AD- Nbnrp1 and BD-PevD1/AD were as negative controls. Y2H assays showed all of the transformants were able to grow on DDO medium (SD medium lacking His and Trp (SD/-His/-Trp), but only the BD-PevD1/AD-Nbnrp1 co-transformed yeast could grow on QDO/A/x-a-gal medium (SD/-Ade/-His/-Ieu/-Trp/AbA/x-a-gal), indicating PevD1 could interact with Nbnrp1 in yeast (**Figure 1C**). BiFC was further performed to verify the interaction between PevD1 and Nbnrp1 in tobacco cells. CFP fluorescence was detected in tobacco cells under laser confocal scanning microscopy, showing the two proteins could interact in tobacco cell (**Figure 1D**).

### The DCD Domain Is Required for Nbnrp1-PevD1 Interaction

**FIGURE 2:**
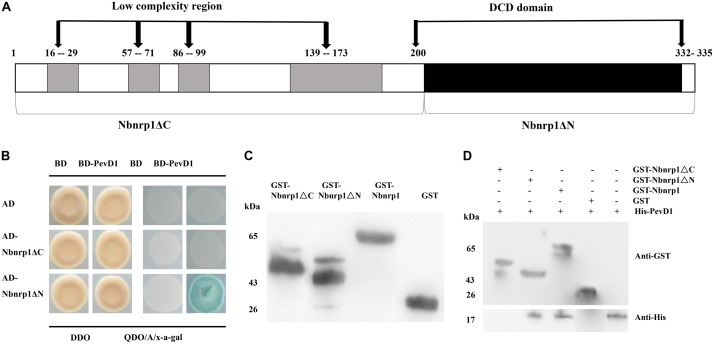
The DCD domain of Nbnrp1 is required for the interaction between PevD1 and Nbnrp1. **(A)** Schematic overview of the domains of Nbnrp1. Open bars indicate coding regions, whereas shaded bars depict the DCD domain. **(B)** Y2H analysis of the interaction between Nbnrp1 deletion mutants and PevD1. The indicated AD and BD constructs were transformed into the Y2H gold yeast strain. The activity of protein–protein interactions were assayed using reporter genes based on growth of transformants on QDO/A/x-α-gal selective medium (SD/-Ade/-His/-Leu/-Trp) medium containing Aureobasidin A and x-α-gal and DDO (SD/-Leu/-Trp) medium. **(C)** Purification and detection of the recombinant proteins. GST-Nbnrp1 (63 kDa), GST-Nbnrp1ΔN (41 kDa) and GST-Nbnrp1ΔC (48 kDa) were expressed in *Escherichia coli* and purified through affinity chromatography and ion exchange chromatography. The purified protein showed corresponding band following SDS-PAGE stained with Coomassie Brilliant Blue R-250. **(D)** GST pull-down assay. The GST and His-PevD1 proteins were used in the negative control, the His-PevD1 protein alone was used in the positive control. The Western-blot shows that the DCD domain is required for the Nbnrp1-PevD1 interaction.

To determine whether the DCD domain of Nbnrp1 involved in the interaction between PevD1 and Nbnrp1, we constructed two Nbnrp1 deletion mutants: Nbnrp1ΔC (residues 1–199), which contained four sequences of low compositional complexity, and Nbnrp1ΔN (residues 200–332), which contained the conserved DCD domain (**Figure 2A**). The Y2H assays showed that only Nbnrp1ΔN could interact with PevD1 in yeast cells (**Figure 2B**). To further confirm the Y2H results, GST pull-down assay was performed. The purified GST-Nbnrp1, GST-Nbnrp1ΔN, GST-Nbnrp1ΔC, and GST proteins were produced in *E. coli* (**Figure 2C**). The GST pull-down assay showed that GST-Nbnrp1 and Nbnrp1ΔN could interact with PevD1 (**Figure 2D**). These results suggested that the DCD domain is required for the Nbnrp1-PevD1 interaction.

### Generation of Nbnrp1-Overexpressing and Nbnrp1-Silence Transgenic Tobacco Plants

**FIGURE 3:**
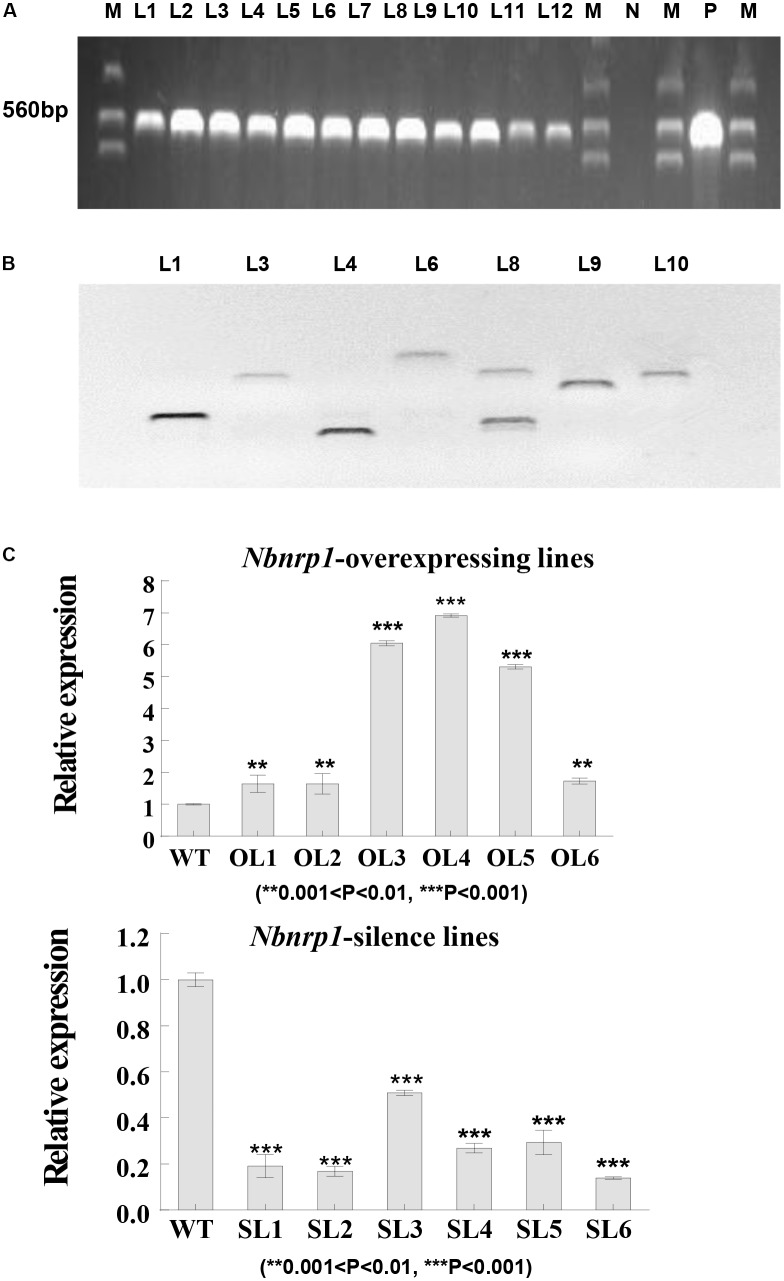
Molecular analysis of transgenic *N. benthamiana*. **(A)** PCR identification of transgenic tobacco plants. M, marker; N, negative control; P, positive control; L1-12, transgenic tobacco lines. **(B)** Southern blot analysis of transgenic tobacco plants (Restriction Enzyme: *BamH* I, *Sal* I, and *EcoR* I). **(C)** The efficiency of *Nbnrp1* overexpressing and the efficiency of *Nbnrp1* silencing: WT, wild type; OL1, OL2, OL3, OL4, OL5, OL6, *Nbnrp1* overexpressing lines; SL1, SL2, SL3, SL4, *Nbnrp1* silence lines. The error bars represent the mean ± SD of three biological replicates. Asterisks indicate a significant difference from the WT by Student’s *t*-test. (^∗∗^0.001 < *P* < 0.01, ^∗∗∗^*P* < 0.001).

To investigate the role of Nbnrp1 in PevD1-induced necrosis and disease resistance, Nbnrp1-overexpressing and Nbnrp1-silence transgenic tobacco plants are generated. The fusion vector was transformed into tobacco via the *Agrobacterium*-mediated method. A specific fragment of the *NTPII* gene (approximately 560 bp in length) was amplified to identify the transgenic lines (**Figure 3A**). Southern blotting showed that the identified transgenic plants carried a single copy according to the protocol (**Figure 3B**). The expression level of *Nbnrp1* in transgenic plants was detected by qPCR, we then selected *Nbnrp1*-overexpressing lines with high Nbnrp1 expression level (OL3 and OL4) and *Nbnrp1*-silence lines with high silence efficiency (SL2 and SL6) for further study (**Figure 3C**). The T3 homozygous were used for PevD1-mediated cell death and the disease resistance assays.

### Effect of *Nbnrp1*-Overexpressing Lines and *Nbnrp1*-Silence Lines on PevD1-Induced Necrosis Response

**FIGURE 4:**
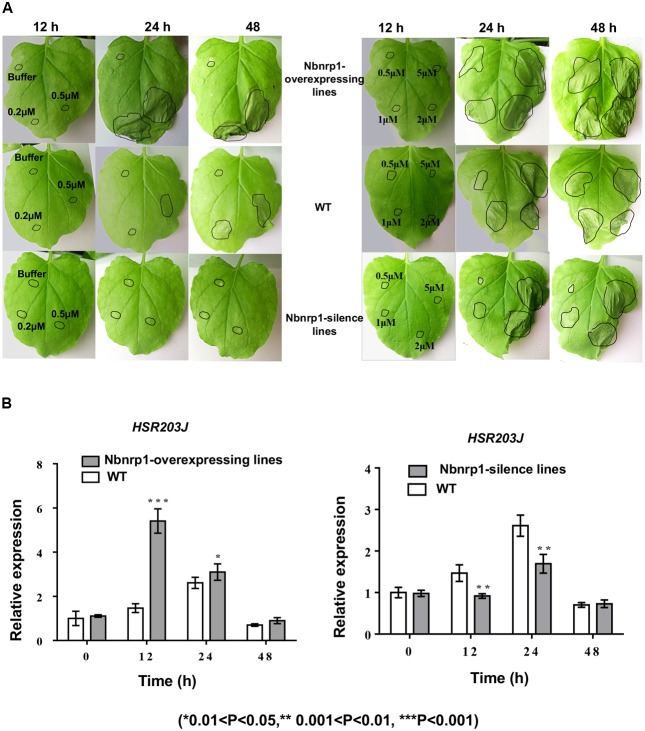
Hypersensitive response (HR) phenotype induced by PevD1 in *N. benthamiana*. **(A)** Necrotic lesions were observed at 12, 24, and 48 h post-PevD1 infiltration. **(B)** Comparison of the expression of the *HSR203J* gene in wild-type, *Nbnrp1*-silence lines and *Nbnrp1*-overexpressing lines at 12, 24, and 48 h induced by PevD1 (^∗^0.01 < *P* < 0.05, ^∗∗^0.001 < *P* < 0.01, ^∗∗∗^*P* < 0.001).

The transgenic and wild type tobacco leaves were infiltrated with 50 μl of PevD1 protein solution in a gradient of concentrations (0, 0.2, 0.5, 1, 2, and 5 μM) to understand the role of Nbnrp1 in PevD1-mediated necrosis, the necrosis was then observed at 12, 24, and 48 h post infiltration. *Nbnrp1*-overexpressing lines appeared HR earlier than wild type plants, and showed more severe necrosis at the same observation period. Likewise, *Nbnrp1*-silence lines showed weakened effect compared with wild type plants (**Figure 4A**). Meanwhile, the transcription level of the HR marker gene *HSR203J* increased in the *Nbnrp1*-overexpressing lines and decreased in the *Nbnrp1*-silence line compared with the wild-type at 12 h after PevD1 infiltration (**Figure 4B**). These results indicated that Nbnrp1 mediates PevD1-induced HR responses.

### *Nbnrp1*-Overexpressing and *Nbnrp1*-Silence Lines Impact PevD1-Induced Disease Resistance

**FIGURE 5:**
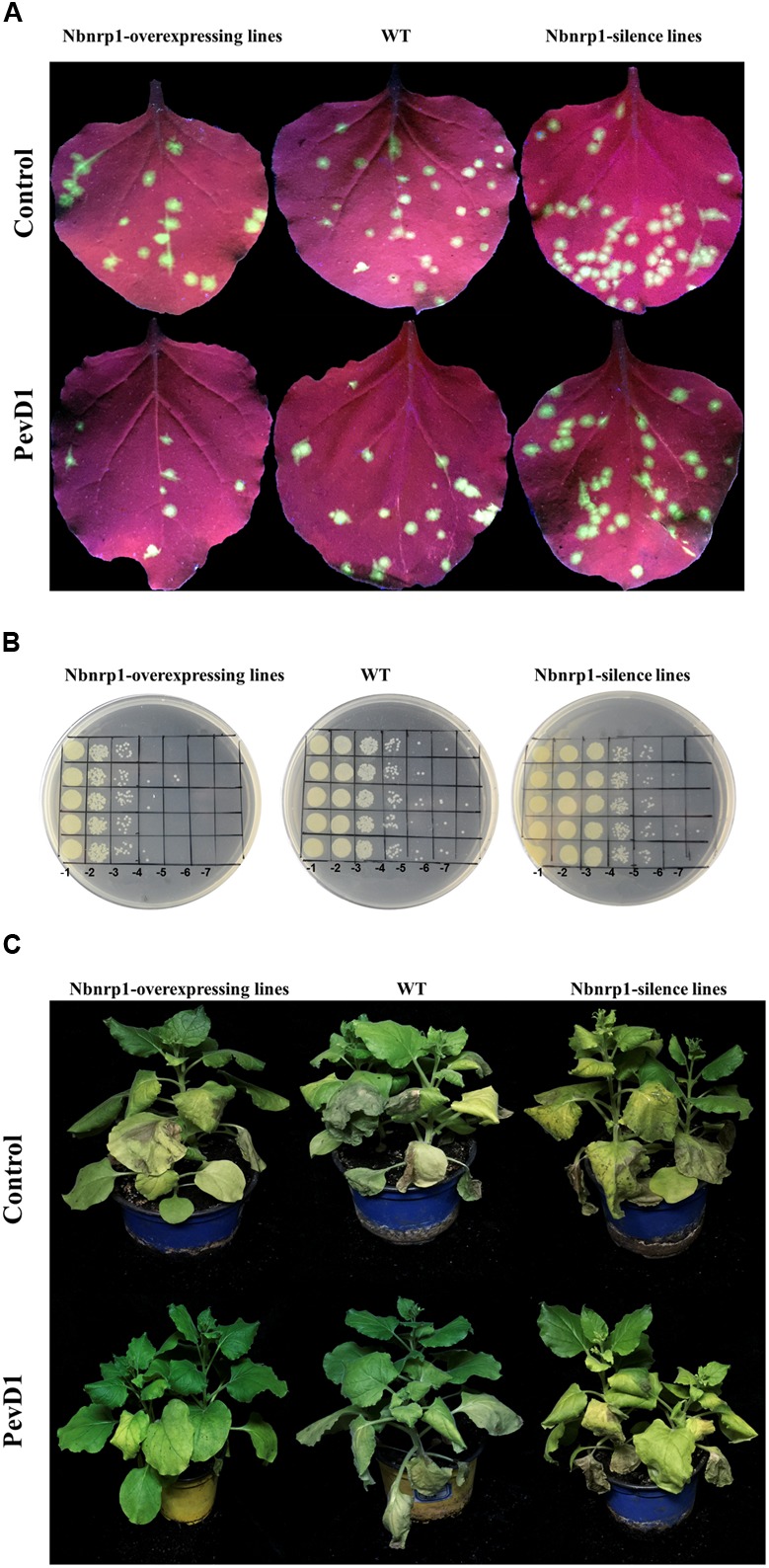
PevD1 induced systemic resistance against infection of TMV-GFP, *P.s.* pv. *tabaci* and *Verticillium dahliae* in transgenic and wild-type *N. benthamiana*. **(A)** The number of TMV-GFP lesions (green fluorescent spots) in systemic leaves of *Nbnrp1*-overexpressing lines, *Nbnrp1*-silence lines and wild type plants. GFP images were obtained under UV illumination at 4 dpi. **(B)** Resistance against *P.s.* pv. *tabaci* in systemic leaves of *Nbnrp1*-overexpressing lines, *Nbnrp1*-silence lines and wild type plants. **(C)** The disease caused by *V. dahliae* strains after treatment of PevD1, BSA was used as negative control.

**FIGURE 6:**
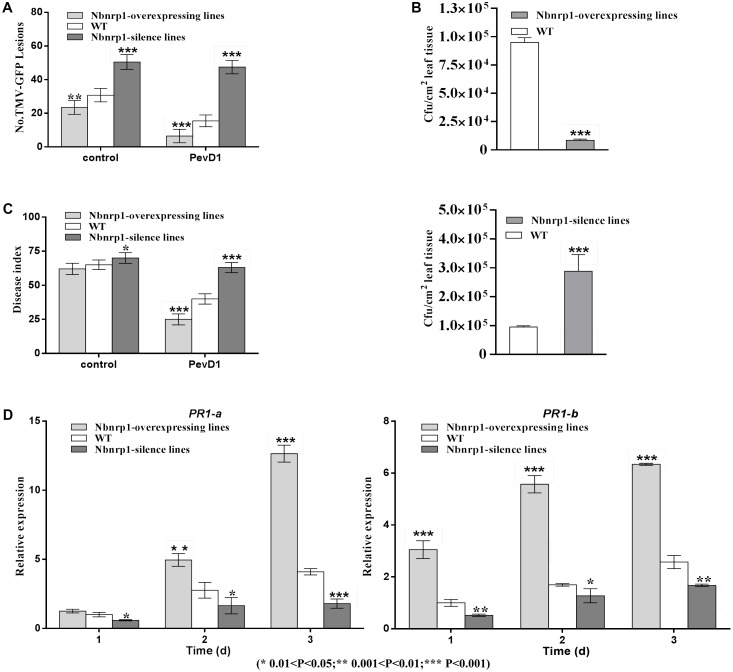
**(A)** The number of GFP puncta in wild-type plants was compared with that in *Nbnrp1*-overexpressing lines and the *Nbnrp1*-silence lines. The data showed significantly difference TMV-GFP infection, being observed at 3 dpi. The results are mean values (±SD) from three independent experiments. **(B)** The number of CFU/cm^2^ in wild-type plants was compared with two mutants and displayed significantly difference infection by *P.s.* pv. *tabaci*, being observed at 3 dpi. **(C)** The disease caused by *V. dahliae* strains as measured by the disease index. Asterisks indicate a significant difference from the WT by Student’s *t*-test. (^∗^0.01 < *P* < 0.05, ^∗∗^0.001 < *P* < 0.01, ^∗∗∗^*P* < 0.001). **(D)** Expression levels of the defense-related genes *PR1-a* and *PR1-b*. *N. benthamiana* leaves were infiltrated with PevD1. At the indicated times, the relative expression levels of *PR1-a* and *PR1-b* genes were measured via qPCR. The samples were normalized against *EF-1a*. Expression levels are represented as the fold change in relation to the wild-type plants. The results are mean values (±SD) from three independent experiments. The statistical analyses were performed using Student’s *t*-test (^∗^0.01 < *P* < 0.05, ^∗∗^0.001 < *P* < 0.01, ^∗∗∗^*P* < 0.001).

The *N. benthamiana Nbnrp1*-overexpressing lines, *Nbnrp1*-silence lines and wild type were inoculated with TMV at 3 days post PevD1 infiltration, respectively. The *Nbnrp1*-overexpressing lines showed enhanced disease resistance against TMV and the number of TMV-GFP lesions in systemic leaves was obviously less than that of wild type plants. The number of TMV lesions was reduced by approximately 41.2% at 4 dpi. But the *Nbnrp1*-silence lines appeared attenuated TMV disease resistance. The number of TMV-GFP lesions in systemic leaves was obviously more than that of wild-type plants, and the number of TMV lesions increased by 71.7% at 4 dpi (**Figures 5A**, **6A**). Furthermore, the *Nbnrp1*-overexpressing lines also exhibited enhanced systemic resistance against *P.s.* pv. *tabaci*, the bacterial counts in the *Nbnrp1*-overexpressing lines were significantly reduced by 91.1% at 3 dpi. While the *Nbnrp1*-silence lines attenuated systemic resistance against *P.s.* pv. *tabaci*. The bacterial counts in the *Nbnrp1*-silence lines were increased by 67% at 3 dpi (**Figures 5B**, **6B**). Besides, the capacity of PevD1 to induce systemic disease resistance against the fungal pathogen *V. dahliae* in *Nbnrp1*-overexpressing lines was significantly higher than that in *Nbnrp1*-silence lines and wild type. Compared with wild type, the disease index displayed a statistically significant decrease in *Nbnrp1*-silence lines (**Figures 5C**, **6C**). These data indicate that Nbnrp1 positively regulates PevD1-induced disease resistance.

To further confirm that the *Nbnrp1*-overexpressing lines and *Nbnrp1*-silence lines impact PevD1-induced disease resistance, the relative expression level of two defense-related genes *PR1-a* and *PR1-b* were detected via qPCR. The relative expression level of *PR1-a* and *PR1-b* were elevated in the *Nbnrp1*-overexpressing lines and declined in the *Nbnrp1*-silence lines compared to wild type plants at 3 days post PevD1 infiltration (**Figure 6D**).

## Discussion

The fungal pathogen *V. dahliae* causes serious yield losses worldwide in extensive plants including cotton and tobacco (Miao et al., 2010). Hundreds of proteins are secreted in *V. dahliae* infection process and contribute to regulate compatible and incompatible plant-pathogen interaction (Ellis et al., 2006). However, how these fungal secretory proteins enter the plant cell is still limited. Some of these proteins have been shown to produce necrosis and induce oxidative burst and phytoalexin accumulation when applied to plants in an isolated form. For example, a 65 kDa glycoprotein from the *V. dahliae* could induce phytoalexin accumulation and oxidative burst (Davis et al., 1998). The fusion protein VdNEP triggered PR gene expression, gossypol and sesquiterpene phytoalexins synthesis (Wang et al., 2004).

We previously characterized a *V. dahliae* new secretory protein PevD1 that could trigger cell death in tobacco as well as a series of defense responses and improve disease resistance to TMV and *V. dahliae* (Wang et al., 2011; Bu et al., 2014). Whereas how PevD1 trigger plant immunity is unknown. The interacting partner is a key player in uncovering defense signal network. Our previous research demonstrated that PevD1 could interact with *Arabidopsis* NRP protein, while NRPs are conserved in plant kingdom. NRPs involved in responsing to different stresses such as salt stress, oxidative stress, mechanical perturbation and pathogen infection (Hoepflinger et al., 2011). NRPs also mediated cell death signaling in ER stress pathway, and further researches showed that DCD domain plays an important role in cell death (Tenhaken et al., 2005; Faria et al., 2011). To investigate that NRP mediated PevD1-induced immune response in *N. benthamiana*, in present research, we cloned homologous gene of *AtNRP* from *N. benthamiana* genome, named as *Nbnrp1* that encode a putative protein of NRP family. Binding assays showed that Nbnrp1 could bind with PevD1 (**Figure 1**) and C-terminal DCD domain of Nbnrp1 was required for PevD1-Nbnrp1 interaction (**Figure 2**).

Nbnrp1 is a putative protein of NRP family in *N. benthamiana* genome and its functions are unclear. Our research showed that *Nbnrp1*-silence plant lines attenuated PevD1-induced necrotic cell death (**Figure 4A**) and the transcription of the HR marker gene *HSR203J* also reduced (**Figure 4B**). Furthermore, the *Nbnrp1*-overexpressing transgenic lines displayed accelerated cell death and elevated transcription of marker gene *HSR203J*. This phenomenon indicated that Nbnrp1 positively regulate PevD1-induced cell death and disease resistance, although detail mechanism is still unknown. It is first report that *N. benthamiana* NRP mediates fungal elicitor protein-triggered plant immunity.

Plant cell death is a conventional indicator of resistance, which based on plant disease resistance genes (*R* genes) recognizing the pathogen avirulence (*Avr*) genes, leading to activate cell death pathways (Heath, 2000). Except for *R* genes, plant immunity components or regulators are determinants of necrotic cell death via the directly or indirectly association with pathogen elicitors/effectors. For example, tomato papain-like cysteine protease (PLCP), C14 target pathogen effectors EPIC1 and EPIC2B secreted from *Phytophthora infestans*, contributed to immunity via participation in cell death signaling. C14 silencing plants showed increased susceptibility to hemibiotrophs of *P. infestans* (Kaschani et al., 2010). In our present research, bioassay showed the *Nbnrp1*-overexpressing lines and *Nbnrp1*-silence lines remarkable influence PevD1-induced necrosis activity and disease resistance against viruses TMV and bacterial pathogen *P.s.* pv. *tabaci*. These results indicated that Nbnrp1 mediate PevD1-induced cell death and resistance in *N. benthamiana*. However, how the Nbnrp1 mediate or regulate cell death signal activated by elicitor PevD1 remains unknown. Whether Nbnrp1 mediate ER-stress cell death also require further exploration.

The structure of PevD1 resembles C2 domain-like structure with a calcium ion bound to the C-terminal acidic pocket and C-terminal 57 amino acid of PevD1 is essential to trigger HR in *N. benthamiana* (Liu et al., 2014; Zhou et al., 2017). Calcium ion (Ca^2+^) plays a crucial role in regulating cellular responses to environmental stresses (Dodds and Rathjen, 2010). Ca^2+^ concentration increases when plant perceived stimulus and activates downstream of Ca^2+^ signal transduction pathways. In view of the above reasons, we speculated that C-terminus of PevD1 maybe contains a key amino acid fragment that responsible to bind partner protein Nbnrp1, which activates MAPK cascade leading to plant immune response. Details of amino acid in PevD1 binding with Nbnrp1 remain need to be investigated further.

Overexpression of NRP in soybean protoplast resulted in chlorophy II reducing, leaf chlorosis and senescence marker gene induction (Costa et al., 2008; Reis et al., 2011). Whereas, there was no abnormal development and leaf chlorosis were observed in *Nbnrp1*-overexpressing lines and *Nbnrp1*-silence lines. We speculated that the function of Nbnrp1 in *N. benthamiana* may be different from that of NRPs in soybean, although they belong to the same family of N-rich proteins and exhibit about 60% similarity. The effect of Nbnrp1 in *N. benthamiana* development needs further research.

## Conclusion

We first identified the PevD1 binding protein Nbnrp1 in *N. benthamiana* and demonstrated that Nbnrp1 mediate PevD1-induced cell death and disease resistance. DCD domain of Nbnrp1 was required for the interaction with PevD1. The Nbnrp1 in *N. benthamiana* transgenic lines obviously impacted PevD1-induced cell death and disease resistance to TMV, bacterial pathogen *P.s.* pv. *tabaci* and fungal pathogen *V. dahliae* compared with the wild type plants. Our results demonstrated that the protein Nbnrp1 positively modulates PevD1-induced immune response in *N. benthamiana*.

## Author Contributions

XY designed the experiments. YL and SC carried out the experiments and wrote the manuscript. XT analyzed the experimental results. YZ, DQ, HZ, LG, and JY assisted the experiments.

## Conflict of Interest Statement

The authors declare that the research was conducted in the absence of any commercial or financial relationships that could be construed as a potential conflict of interest.

## Footnotes

1 https://solgenomics.net/

## References

[B1] Akira S. Uematsu S. Takeuchi O. (2006). Pathogen recognition and innate immunity. *Cell* 124 783–801. 10.1016/j.cell.2006.02.015 16497588

[B2] Boller T. Felix G. (2009). A renaissance of elicitors: perception of microbe-associated molecular patterns and danger signals by pattern-recognition receptors. *Annu. Rev. Plant Biol.* 60 379–406. 10.1146/annurev.arplant.57.032905.105346 19400727

[B3] Bu B. Qiu D. Zeng H. Guo L. Yuan J. Yang X. (2014). A fungal protein elicitor PevD1 induces Verticillium wilt resistance in cotton. *Plant Cell Rep.* 33 461–470. 10.1007/s00299-013-1546-7 24337817

[B4] Chisholm S. T. Coaker G. Day B. Staskawicz B. J. (2006). Host-Microbe interactions: shaping the evolution of the plant immune response. *Cell* 124 803–814. 10.1016/j.cell.2006.02.008 16497589

[B5] Costa M. D. L. Reis P. A. B. Valente M. A. S. Irsigler A. S. T. Carvalho C. M. Loureiro M. E. (2008). A new branch of endoplasmic reticulum stress signaling and the osmotic signal converge on plant-specific asparagine-rich proteins to promote cell death. *J. Biol. Chem.* 283 20209–20219. 10.1074/jbc.M802654200 18490446

[B6] Dangl J. L. Jones J. D. (2001). Plant pathogens and integrated defence responses to infection. *Nature* 411 826–833. 10.1038/35081161 11459065

[B7] Davis D. A. Low P. S. Heinstein P. (1998). Purification of a glycoprotein elicitor of phytoalexin formation from *Verticillium dahliae*^∗^. *Physiol. Mol. Plant Pathol.* 52 259–273. 10.1006/pmpp.1998.0150

[B8] Dean J. D. Goodwin P. H. Hsiang T. (2002). Comparison of relative RT-PCR and northern blot analyses to measure expression of β-1,3-glucanase in *Nicotiana benthamiana* infected with *Colltotrichum destructivum*. *Plant Mol. Biol. Rep.* 20 347–356. 10.1007/bf02772122

[B9] Desaki Y. Miya A. Venkatesh B. Tsuyumu S. Yamane H. Kaku H. (2006). Bacterial lipopolysaccharides induce defense responses associated with programmed cell death in rice cells. *Plant Cell Physiol.* 47 1530–1540. 10.1093/pcp/pcl019 17018557

[B10] Dodds P. N. Rathjen J. P. (2010). Plant immunity: towards an integrated view of plant-pathogen interactions. *Nat. Rev. Genet. Nat.* 11 539–548. 10.1038/nrg2812 20585331

[B11] Ellis J. Catanzariti A.-M. Dodds P. (2006). The problem of how fungal and oomycete avirulence proteins enter plant cells. *Trends Plant Sci.* 11 61–63. 10.1016/j.tplants.2005.12.008 16406302

[B12] Faria J. A. Q. A. Reis P. A. B. Reis M. T. B. Rosado G. L. Pinheiro G. L. Mendes G. C. (2011). The NAC domain-containing protein, GmNAC6, is a downstream component of the ER stress- and osmotic stress-induced NRP-mediated cell-death signaling pathway. *BMC Plant Biol.* 11:129. 10.1186/1471-2229-11-129 21943253 PMC3193034

[B13] Heath M. C. (2000). Hypersensitive response-related death. *Plant Mol. Biol.* 44 321–334. 10.1023/A:1026592509060 11199391

[B14] Hoepflinger M. C. Pieslinger A. M. Tenhaken R. (2011). Investigations on N-rich protein (NRP) of *Arabidopsis thaliana* under different stress conditions. *Plant Physiol. Biochem.* 49 293–302. 10.1016/j.plaphy.2011.01.005 21277785

[B15] Horsch R. B. (1985). A simple and general method for transferring genes into plants. *Science* 227 1229–1231. 10.1126/science.227.4691.1229 17757866

[B16] Jin Y. Ma D. Dong J. Li D. Deng C. Jin J. (2007). The HC-Pro protein of potato virus Y interacts with NtMinD of tobacco. *Mol. Plant Microbe Interact.* 20 1505–1511. 10.1094/MPMI-20-12-1505 17990958

[B17] Kaschani F. Shabab M. Bozkurt T. Shindo T. Schornack S. Gu C. (2010). An effector-targeted protease contributes to defense against *Phytophthora infestans* and is under diversifying selection in natural hosts. *Plant Physiol.* 154 1794–1804. 10.1104/pp.110.158030 20940351 PMC2996022

[B18] Katagiri F. Tsuda K. (2010). Understanding the plant immune system. *Mol. Plant Microbe Interact.* 23 1531–1536. 10.1094/MPMI-04-10-0099 20653410

[B19] Li H. Y. Guo Z. F. Zhu Y. X. (1998). Molecular cloning and analysis of a pea cDNA that is expressed in darkness and very rapidly induced by gibberellic acid. *Mol. Gen. Genet.* 259 393–397. 10.1007/s004380050828 9790595

[B20] Liu M. Khan N. U. Wang N. Yang X. Qiu D. (2016). The protein elicitor PevD1 enhances resistance to pathogens and promotes growth in *Arabidopsis*. *Int. J. Biol. Sci.* 12 931–943. 10.7150/ijbs.15447 27489497 PMC4971732

[B21] Liu W. Zeng H. Liu Z. Yang X. Guo L. Qiu D. (2014). Mutational analysis of the *Verticillium dahliae* protein elicitor PevD1 identifies distinctive regions responsible for hypersensitive response and systemic acquired resistance in tobacco. *Microbiol. Res.* 169 476–482. 10.1016/j.micres.2013.08.001 24080193

[B22] Liu Y. Schiff M. Marathe R. Dinesh-Kumar S. P. (2002). Tobacco Rar1 EDS1 and NPR1/NIM1 like genes are required for N-mediated resistance to tobacco mosaic virus. *Plant J.* 30 415–429. 10.1046/j.1365-313X.2002.01297.x 12028572

[B23] Ludwig A. A. Tenhaken R. (2001). A new cell wall located N-rich protein is strongly induced during the hypersensitive response in *Glycine Max* L. *J. Plant Pathol.* 107 323–336. 10.1023/A:1011202225323

[B24] Miao W. Wang X. Li M. Song C. Wang Y. Hu D. (2010). Genetic transformation of cotton with a harpin-encoding gene *hpa Xoo* confers an enhanced defense response against different pathogens through a priming mechanism. *BMC Plant Biol.* 10:67. 10.1186/1471-2229-10-67 20398293 PMC3095341

[B25] Mur L. A. J. Kenton P. Lloyd A. J. Ougham H. Prats E. (2008). The hypersensitive response; the centenary is upon us but how much do we know? *J. Exp. Bot.* 59 501–520. 10.1093/jxb/erm239 18079135

[B26] Pennell R. I. Lamb C. (1997). Programmed cell death in plants. *Plant Cell* 9 1157–1168. 10.1105/tpc.9.7.1157 12237381 PMC156988

[B27] Reis P. A. A. Rosado G. L. Silva L. A. C. Oliveira L. C. Oliveira L. B. Costa M. D. L. (2011). The binding protein BiP attenuates stress-induced cell death in soybean via modulation of the n-rich protein-mediated signaling pathway. *Plant Physiol.* 157 1853–1865. 10.1104/pp.111.179697 22007022 PMC3327224

[B28] Sambrook J. Russell D. W. (2004). Detection of protein-protein interactions using the GST fusion protein pull-down technique. *Nat. Methods* 1 275–276. 10.1038/nmeth1204-275

[B29] Schmittgen T. D. Livak K. J. (2008). Analyzing real-time PCR data by the comparative CT method. *Nat. Protoc.* 3 1101–1108. 10.1038/nprot.2008.73 18546601

[B30] Schrader S. Kaldenhoff R. Richter G. (1997). Expression of novel genes during somatic embryogenesis of suspension-cultured carrot cells (*Daucus carota*). *J. Plant Physiol.* 150 63–68. 10.1016/s0176-1617(97)80182-4

[B31] Shi F. Dong Y. Zhang Y. Yang X. Qiu D. (2017). Overexpression of the PeaT1 elicitor gene from *Alternaria tenuissima* improves drought tolerance in rice plants *via* interaction with a myo-inositol oxygenase. *Front. Plant Sci.* 8:970. 10.3389/fpls.2017.00970 28649255 PMC5465376

[B32] Tenhaken R. Doerks T. Bork P. (2005). DCD - a novel plant specific domain in proteins involved in development and programmed cell death. *BMC Bioinformatics* 6:169. 10.1186/1471-2105-6-169 16008837 PMC1182354

[B33] Thomma B. P. Nürnberger T. Joosten M. H. A. J. (2011). Of PAMPs and effectors: the blurred PTI-ETI dichotomy. *Plant Cell* 23 4–15. 10.1105/tpc.110.082602 21278123 PMC3051239

[B34] Tsuda K. Katagiri F. (2010). Comparing signaling mechanisms engaged in pattern-triggered and effector-triggered immunity. *Curr. Opin. Plant Biol.* 13 459–465. 10.1016/j.pbi.2010.04.006 20471306

[B35] Waadt R. Schmidt L. K. Lohse M. Hashimoto K. Bock R. Kudla J. (2008). Multicolor bimolecular fluorescence complementation reveals simultaneous formation of alternative CBL/CIPK complexes in planta. *Plant J.* 56 505–516. 10.1111/j.1365-313X.2008.03612.x 18643980

[B36] Walter M. Chaban C. Schütze K. Batistic O. Weckermann K. Näke C. (2004). Visualization of protein interactions in living plant cells using bimolecular fluorescence complementation. *Plant J.* 40 428–438. 10.1111/j.1365-313X.2004.02219.x 15469500

[B37] Wang B. Yang X. Zeng H. Liu H. Zhou T. Tan B. (2011). The purification and characterization of a novel hypersensitive-like response-inducing elicitor from Verticillium dahliae that induces resistance responses in tobacco. *Appl. Microbiol. Biotechnol.* 93 191–201. 10.1007/s00253-011-3405-1 21691787

[B38] Wang J. Cai Y. Gou J. Mao Y. Xu Y. Jiang W. (2004). VdNEP, an elicitor from *Verticillium dahliae*, induces cotton plant wilting. *Appl. Environ. Microbiol.* 70 4989–4995. 10.1128/AEM.70.8.4989-4995.2004 15294839 PMC492334

[B39] Zhang Y. Gao Y. Liang Y. Dong Y. Yang X. Yuan J. (2017a). The *Verticillium dahliae* SnodProt1-Like protein VdCP1 contributes to virulence and triggers the plant immune system. *Front. Plant Sci.* 8:175. 10.3389/fpls.2017.01880 29163605 PMC5671667

[B40] Zhang Y. Liang Y. Dong Y. Gao Y. Yang X. Yuan J. (2017b). The *Magnaporthe oryzae* Alt A 1-like protein MoHrip1 binds to the plant plasma membrane. *Biochem. Biophys. Res. Commun.* 492 55–60. 10.1016/j.bbrc.2017.08.039 28807829

[B41] Zhang Y. Liang Y. Qiu D. Yuan J. Yang X. (2017c). Comparison of cerato-platanin family protein BcSpl1 produced in *Pichia pastoris* and *Escherichia coli*. *Protein Expr. Purif.* 136 20–26. 10.1016/j.pep.2017.06.004 28606662

[B42] Zhang Y. Zhang Y. Qiu D. Zeng H. Guo L. Yang X. (2015). BcGs1 a glycoprotein from *Botrytis cinerea*, elicits defence response and improves disease resistance in host plants. *Biochem. Biophys. Res. Commun.* 457 627–634. 10.1016/j.bbrc.2015.01.038 25613865

[B43] Zhou R. Zhu T. Han L. Liu M. Xu M. Liu Y. (2017). The asparagine-rich protein NRP interacts with the *Verticillium* effector PevD1 and regulates the subcellular localization of cryptochrome 2. *J. Exp. Bot.* 68 3427–3440. 10.1093/jxb/erx192 28633330

