# Supplementary material for: An Asparagine-Rich Protein Nbnrp1 Modulate Verticillium dahliae Protein PevD1-Induced Cell Death and Disease Resistance in Nicotiana benthamiana
Source: Front Plant Sci. 2018 Mar 7;9:303. doi: 10.3389/fpls.2018.00303 (PMC5846053; doi:10.3389/fpls.2018.00303)
Supplement: Supplementary file 1 [file Table_1.DOCX]

**Table S1. Primers used for PCR in this study**

| **Gene name** | **Forward primer (5’ to 3’ )** | **Reverse primer (5’ to 3’)** |
| --- | --- | --- |
| **Nbnrp1** | **ATGGAGAACAACAATCAATCATC** | **TTAAGGAAAGCGGGATTCACCAGG** |
| **GST-Nbnrp1** | **CGGGATCCATGGAGAACAACAATC** | **GCGTCGACTTAGTTCTTGTTCTCTTCAA** |
| **GST-Nbnrp1ΔN** | **CGCGGATCCGAAACTGTTGGTGGTTAT** | **ACGCGTCGACTTAGTTCTTGTTCTCTTC** |
| **GST-Nbnrp1ΔC** | **CG GGATCC ATGGAGAACAACAATC** | **GCGTCGACTTAATTTCTTGGCAAAGATTCA** |
| **PBI121-Nbnrp1** | **AGAACACGGGGGACTCTATGGAGAACAACAATCAATCATC** | **CATAAGGGACTGACCACCCGGTTAGTTCTTGTTCTCTTCAAAAA** |
| **Sense-Nbnrp1ΔN** | **GAAGATCTGAATCTTTGCCAAGAA** | **GAAGATCTGTTCTTGTTCTCTTCAAAA** |
| **Anti-sense-Nbnrp1ΔN** | **CGGGATCCGTTCTTGTTCTCTTCAAAAA** | **GCGTCGACGAATCTTTGCCAAGAA** |
